# Elevated triglyceride-glucose index predicts mortality following endovascular abdominal aortic aneurysm repair

**DOI:** 10.3389/fnut.2023.1116425

**Published:** 2023-02-13

**Authors:** Tan Li, Chao Yang, Jun Yang, Jingjing Jing, Chunyan Ma

**Affiliations:** ^1^Department of Cardiovascular Ultrasound, The First Hospital of China Medical University, Shenyang, China; ^2^Clinical Medical Research Center of Imaging in Liaoning Province, The First Hospital of China Medical University, Shenyang, China; ^3^Department of Burns, Trauma Center, The First Hospital of China Medical University, Shenyang, China; ^4^Tumor Etiology and Screening Department of Cancer Institute and General Surgery, The First Hospital of China Medical University, Shenyang, China

**Keywords:** triglyceride, glucose, abdominal aortic aneurysm, biomarker, mortality

## Abstract

**Background:**

Triglyceride-glucose (TyG) index has been increasingly studied as a simple and reliable predictor of adverse events of some cardiovascular disorders. However, its prognostic effect on postoperative outcomes in patients with abdominal aortic aneurysm (AAA) is still unknown. The current study aimed to explore the potential role of TyG index in predicting mortality of AAA patients following endovascular aneurysm repair (EVAR).

**Materials and methods:**

This retrospective cohort study analyzed the preoperative TyG index in a total of 188 AAA patients who underwent EVAR with the follow-up of 5 years. Data were analyzed with SPSS software Version 23.0. Association between the TyG index and all-cause mortality was evaluated using Cox regression models and Kaplan-Meier method.

**Results:**

Cox regression analyses showed that per 1-unit increment of TyG index was significantly associated with an increased risk of postoperative 30-day, 1-year, 3-year, and 5-year mortality, even after adjustment for potential confounders (all *P*<0.05). Kaplan-Meier analysis suggested that patients with high TyG index (≥8.68) had a worse overall survival (*P* = 0.007).

**Conclusion:**

The elevated TyG index could be a promising predictive factor of postoperative mortality in AAA patients after EVAR.

## Introduction

Abdominal aortic aneurysm (AAA) is an irreversible progressive widening of the abdominal aorta and endovascular aneurysm repair (EVAR) intervention is regarded as a safe and effective method of treating AAA (1). Reliable preoperative risk factor detection makes it possible to early predict poor outcomes of AAA patients after surgery in clinical practice. The triglyceride-glucose (TyG) index, a product of fasting triglyceride (TG) and fasting plasma glucose (FPG), is considered to be a simple, cost-effective, valid and reliable surrogate marker for insulin resistance (IR) (2, 3). IR plays a crucial role in the pathogenesis of cardiovascular disease and has been established as a predictor of elevated mortality (4). Recently, several investigations discovered that the high TyG index might be an independent risk factor for all-cause mortality in patients with a variety of cardiovascular disorders (5, 6). For example, acute myocardial infarction and coronary angiography patients with a high TyG index owned a higher risk of all-cause mortality (5). Survival of AAA patients following EVAR may be influenced by age, some inflammatory biomarkers such as white blood cell (WBC) and C-reactive protein (CRP), and comorbidities like diabetes, renal insufficiency and stroke (7, 8). However, the predictive value of TyG index on early and late mortality of AAA patients after EVAR surgery remains unclear.

In this research, we intended to examine whether there was an association between the TyG index and all-cause mortality in AAA patients who received EVAR treatment. Our results may provide ideas for improving AAA risk stratification and clinical decision-making.

## Materials and methods

This retrospective cohort study included 188 AAA patients undergoing EVAR in the First Hospital of China Medical University between February 2015 and November 2017. All patients were diagnosed by computed tomography angiography and successfully followed up for 5 years with complete preoperative fasting TG and FPG levels obtained upon hospital admission. The TyG index was calculated with the following formula: TyG index = Ln [fasting TG (mg/dL) × FPG (mg/dL)/2] (9). The subjects with previous aortic surgery, congenital disorder, malignant tumor, hematological disease or unavailable survival time were excluded. Baseline demographics, clinical information and laboratory data were obtained from hospital electronic medical records. A clinical follow-up was performed *via* telephone or medical records, and the primary outcomes were all-cause mortality during the follow-up period (30 days, 1 year, 3 years, and 5 years after EVAR). We completed the last follow-up visits on November 8, 2022. The study was approved by the Ethics Committee of the First Hospital of China Medical University (Shenyang, China).

Analyses were conducted with SPSS software Version 23.0. Categorical variables were reported in numbers and percentages and continuous variables were expressed as mean ± standard deviation. The differences of TyG index between groups were analyzed using Student's *t*-test. Univariate Cox regression analyses were utilized to identify the factors associated with mortality and variables with univariate *P* < 0.10 were included in the multivariate model for adjustment. We applied the unadjusted and adjusted Cox proportional hazards model to estimate the correlation between TyG index and all-cause mortality, and results were described by hazard ratios (HRs) with 95% confidence intervals (CIs). The optimal cut-point for the TyG index was determined based upon receiver operating characteristic (ROC) analysis. Kaplan-Meier survival analysis with the log-rank test was carried out to estimate the cumulative probability of survival. The statistical significance was defined as a *P*-value (two-sided) < 0.05.

## Results

During the follow-up period of 5 years, a total of 42 patients died with an overall mortality rate of 22.3%. Patients who died had a higher preoperative TyG index than those who survived (8.88 ± 0.80 vs. 8.56 ± 0.59, *P* = 0.004). As presented in Table 1, body mass index (BMI), heart rate, diabetes, WBC and CRP were possible risk factors for 30-day mortality in AAA patients after EVAR. Meanwhile, age, stroke, renal insufficiency, diabetes, WBC and CRP might be related to 1-year mortality, and age, diabetes, WBC, D-dimer and CRP were potential confounding factors for 3- and 5- year mortality.

**Table 1:** Univariate Cox regression analyses of factors associated with mortality following EVAR for AAA during follow-up.

**Variable**	**AAA patients (*n* = 188)**	**30-day mortality (*****n*** = **10)**		**1-year mortality (*****n*** = **25)**		**3-year mortality (*****n*** = **37)**		**5-year mortality (*****n*** = **42)**	
		**HR (95% CI)**	***P*** **value**	**HR (95% CI)**	***P*** **value**	**HR (95% CI)**	***P*** **value**	**HR (95% CI)**	***P*** **value**
Age, years	67.04 ± 10.24	1.017 (0.953–1.085)	0.621	1.051 (1.003–1.102)	0.038	1.038 (0.999–1.079)	0.058	1.048 (1.009–1.089)	0.014
Male, n (%)	152 (80.9%)	0.944 (0.192–4.649)	0.944	0.939 (0.329–2.698)	0.908	0.825 (0.341–1.997)	0.670	0.832 (0.357–1.941)	0.670
BMI, kg/m^2^	23.73 ± 3.00	1.319 (1.054–1.651)	0.016	1.100 (0.956–1.265)	0.185	1.093 (0.969–1.232)	0.149	1.067 (0.952–1.197)	0.265
Heart rate, bmp	77.67 ± 11.42	1.047 (0.995–1.102)	0.075	1.013 (0.977–1.050)	0.493	1.002 (0.971–1.034)	0.880	0.996 (0.966–1.026)	0.771
Smoking, n (%)	71 (37.8%)	0.905 (0.247–3.325)	0.881	0.747 (0.304–1.833)	0.524	1.157 (0.555–2.411)	0.698	1.018 (0.502–2.065)	0.960
Drinking, n (%)	37 (19.7%)	1.021 (0.208–5.024)	0.979	0.750 (0.241–2.336)	0.620	0.749 (0.287–1.955)	0.555	0.773 (0.313–1.912)	0.578
Coronary artery disease, n (%)	42 (22.3%)	0.863 (0.176–4.225)	0.855	1.114 (0.414–2.997)	0.831	0.949 (0.397–2.268)	0.907	1.316 (0.595–2.912)	0.497
Stroke, n (%)	24 (12.8%)	3.204 (0.769–13.350)	0.110	2.544 (0.899–7.200)	0.079	1.430 (0.524–3.900)	0.485	1.518 (0.583–3.949)	0.393
Peripheral arterial disease, n (%)	52 (27.7%)	0.640 (0.131–3.118)	0.581	1.921 (0.802–4.602)	0.143	1.556 (0.722–3.350)	0.259	1.230 (0.581–2.603)	0.589
Chronic obstructive pulmonary disease, n (%)	14 (7.4%)	1.410 (0.166–12.007)	0.753	0.481 (0.060–3.845)	0.490	1.123 (0.297–4.248)	0.864	0.944 (0.251–3.553)	0.932
Renal insufficiency, n (%)	19 (10.1%)	2.368 (0.465–12.061)	0.299	2.661 (0.866–8.177)	0.088	2.055 (0.724–5.829)	0.176	1.705 (0.606–4.800)	0.312
Hypertension, n (%)	135 (71.8%)	1.606 (0.330–7.823)	0.557	1.283 (0.482–3.413)	0.618	1.539 (0.653–3.625)	0.324	1.582 (0.698–3.582)	0.272
Diabetes, n (%)	31 (16.5%)	5.846 (1.581–21.613)	0.008	4.508 (1.793–11.335)	0.001	2.771 (1.187–6.468)	0.018	2.235 (0.971–5.148)	0.059
Dyslipidemia, n (%)	116 (61.7%)	1.396 (0.263–7.411)	0.695	0.623 (0.253–1.535)	0.304	0.867 (0.401–1.875)	0.717	0.981 (0.468–2.056)	0.960
WBC, × 10^9^/L	6.99 ± 2.60	1.251 (1.052–1.487)	0.011	1.162 (1.012–1.333)	0.033	1.215 (1.066–1.384)	0.003	1.179 (1.040–1.337)	0.010
D-dimer, ug/ml	2.77 ± 3.58	0.955 (0.765–1.193)	0.687	1.040 (0.938–1.153)	0.454	1.106 (1.014–1.207)	0.023	1.100 (1.009–1.198)	0.030
CRP, mg/L	23.54 ± 44.82	1.010 (1.001–1.019)	0.027	1.008 (1.001–1.016)	0.029	1.015 (1.006–1.023)	<0.001	1.013 (1.005–1.021)	0.001
Aneurysm diameter, cm	5.55 ± 1.32	1.358 (0.886–2.082)	0.160	0.962 (0.696–1.330)	0.815	1.037 (0.792–1.359)	0.790	1.041 (0.805–1.347)	0.759

EVAR, Endovascular aneurysm repair; AAA, Abdominal aortic aneurysm; HR, Hazard ratio; CI, Confidence interval; BMI, Body mass index; WBC, White blood cell; CRP, C-reactive protein.

The significant association between the TyG index and postoperative mortality was found in both univariate and adjusted regression models (Table 2). Under Cox regression with multiple adjustments, per 1-unit increment of TyG index remained to have a relationship with a 3.015-, 2.848-, 2.524-, and 2.560-fold greater risk for 30-day, 1-year, 3-year, and 5-year mortality, respectively (all *P* < 0.05; Table 2).

**Table 2:** The association between the TyG index and postoperative mortality of AAA patients.

	**HR (95% CI)**	***P* value**
**Univariate analysis**		
30-day mortality	3.019 (1.447–6.297)	0.003
1-year mortality	2.379 (1.330–4.256)	0.003
3-year mortality	2.076 (1.223–3.524)	0.007
5-year mortality	2.043 (1.218–3.427)	0.007
**Multivariate analysis**		
30-day mortality^a^	3.015 (1.269–7.163)	0.012
1-year mortality^b^	2.848 (1.369–5.925)	0.005
3-year mortality^c^	2.524 (1.294–4.922)	0.007
5-year mortality^c^	2.560 (1.381–5.083)	0.003

a Adjusted for BMI, heart rate, diabetes, WBC, and CRP.

b Adjusted for age, stroke, renal insufficiency, diabetes, WBC, and CRP.

c Adjusted for age, diabetes, WBC, D-dimer, and CRP.

TyG, Triglyceride-glucose; AAA, Abdominal aortic aneurysm; HR, Hazard ratio; CI, Confidence interval; BMI, Body mass index; WBC, White blood cell; CRP, C-reactive protein.

**Figure 1:**
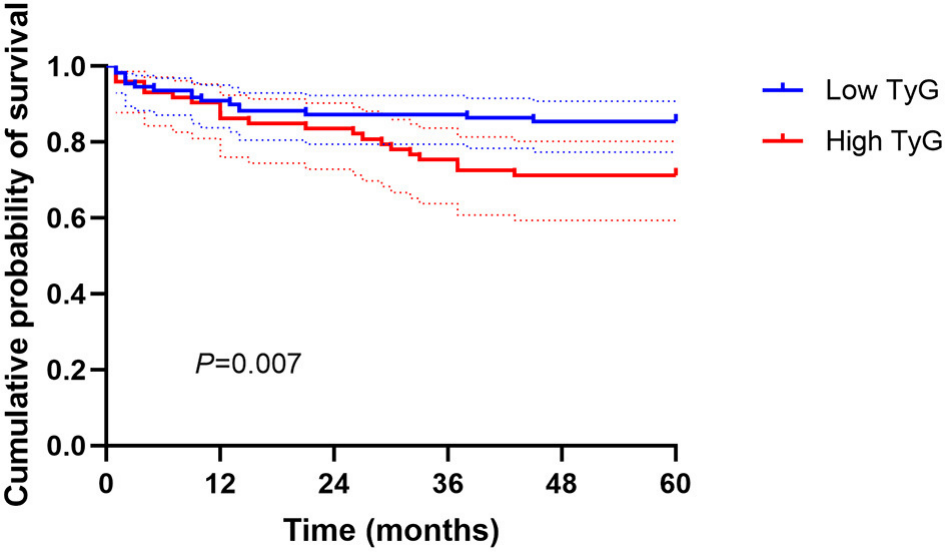
Kaplan-Meier curves of the cumulative survival probability according to TyG index above and below a cutoff value of 8.68.

The ROC analysis determined that the predictive cutoff value of the TyG index for overall mortality was 8.68, yielding an area under the curve of 0.626 (95% CI = 0.529–0.723, *P* = 0.013). Kaplan-Meier analysis indicated that patients with high TyG index (≥8.68) suffered worse cumulative survival than those with low TyG index (<8.68; *P* = 0.007; Figure 1).

## Discussion

To the best of our knowledge, this was the first analysis to assess the effect of TyG index on clinical outcomes in AAA patients following EVAR, with a focus on both short-term and long-term mortality. According to the results, we demonstrated that an increase in the TyG index was significantly correlated with a higher risk of 30-day, 1-year, 3-year, and 5-year mortality, independent of potential risk factors. The survival probability in the TyG index ≥ 8.68 group was markedly lower than that in the TyG index <8.68 group. These findings highlight the usefulness of this simple and easy-to-calculate index to early identify AAA subjects at high risk of developing severe condition after EVAR therapy.

It should be noted that about 80.9% of AAA patients in the present cohort are males, which may be ascribed to the fact that AAA is a complex condition primarily affecting older men with an approximate 6:1 prevalence in males relative to females, and various studies support the protective role of estrogen against AAA (10, 11). Currently, the differential of the TyG index has come into focus to elucidate its role in the prognosis of patients with several forms of cardiovascular disease. It has been reported that higher TyG values were linked with an enhanced risk of adverse cardiovascular events in ST-elevation myocardial infarction patients who underwent percutaneous coronary intervention (12). Zhou et al. found that a TyG index >8.7 was connected to a higher risk of all-cause mortality in individuals with ischemic stroke (6). Notably, we discovered a similar threshold value of around 8.7 for elevated risk of mortality in patients after AAA repair.

Although the exact mechanism underlying the interaction between the TyG index and poor outcomes is not fully understood, it may be explained as follows. First, TyG index is a reliable indicator to evaluate IR. Previous evidence has suggested that IR can favor AAA initiation and progression (13). Moreover, IR usually leads to increased systemic and tissue inflammation, oxidative stress, endothelial dysfunction and renin-angiotensin-aldosterone system activation, thereby resulting in cell damage and affecting the longevity of individual's life (3, 14, 15). Second, TyG is usually associated with lipid and glucose metabolism disturbance, reflecting well the fat toxicity and sugar toxicity that can adversely affect cardiovascular outcomes (16, 17). In addition, a higher TyG index is independently related to the occurrence and development of arterial stiffness, nephric microvascular damage and several diseases including hypertension, diabetes, stroke, coronary artery disease, non-alcoholic fatty liver and malignancy, which are identified as risk factors for early and late mortality in AAA patients after EVAR (4, 17–19). Therefore, it seems reasonable to propose that AAA subjects with higher TyG levels are more likely to experience a course of vascular and organ impairment, contributing to an increased risk of all-cause mortality after EVAR. In clinical practice, our findings may guide clinicians to normalize the preoperative glucose or triglyceride under a target range, which is conducive to a good prognosis for patients with AAA repair.

Some limitations should be acknowledged. First, the TyG index was only collected at baseline and its changes during follow-up were not measured. Second, some influence of selection bias could not be excluded. Third, this was a single-center study based on a relative small cohort. Larger multicenter studies are necessary to confirm our results and define the causal effects of elevated TyG with mortality following EVAR for AAA.

In summary, TyG index could serve as a novel independent predictor for all-cause mortality in AAA patients after EVAR surgery and we also determined a specific cutoff value of TyG relevant to increased mortality. The detection of the TyG index may benefit the early risk stratification and clinical intervention to prevent postoperative all-cause death events.

## Data availability statement

The original contributions presented in the study are included in the article/supplementary material, further inquiries can be directed to the corresponding authors.

## Ethics statement

The studies involving human participants were reviewed and approved by the Ethics Committee of the First Hospital of China Medical University (Shenyang, China). Written informed consent for participation was not required for this study in accordance with the national legislation and the institutional requirements.

## Author contributions

TL, JJ, and CM: designed the study and interpreted the findings and revised the manuscript. TL: analyzed data and wrote the manuscript. TL and CY: carried out the study. CY and JY: did clinical data collection. All authors contributed to the article and approved the submitted version.
